# Relationship Between Thermal Environment and Welfare Indicators of Laying Hens: A Canonical Correlation Approach

**DOI:** 10.1111/asj.70171

**Published:** 2026-03-13

**Authors:** Carolina de Oliveira Marques de Souza, Silvana Cavalcante Bastos Leite, Maria Rogervânia Silva de Farias, Angela Maria de Vasconcelos, Luiz Paulo Fávero, Aérica Cirqueira Nazareno, Laura Bertolaso de Vecchi, Robson Mateus Freitas Silveira

**Affiliations:** ^1^ MBA in Data Science, “Luiz de Queiroz” Agriculture College (ESALQ) University of São Paulo (USP) Piracicaba São Paulo Brazil; ^2^ Department of Animal Science University of Vale Acaraú Sobral Sobral Ceará Brazil; ^3^ Department of Animal Science Federal University of Viçosa Viçosa Brazil; ^4^ Faculty of Economics, Administration, Accounting and Actuarial Science University of São Paulo São Paulo Brazil; ^5^ Center for Nuclear Energy in Agriculture (CENA/USP) University of São Paulo Piracicaba São Paulo Brazil; ^6^ Environment Livestock Research Group (NUPEA), “Luiz de Queiroz” Agriculture College (ESALQ), Department of Biosystems Engineering University of São Paulo (USP) Piracicaba São Paulo Brazil

**Keywords:** animal production, behavior, heat stress, multivariate

## Abstract

Adaptation of laying hens to heat stress involves variables and multiple mechanisms, which are complex and interconnected by linear responses and interactions. Here, we compare the Pearson correlation method and canonical correlation to evaluate relationships between thermoregulatory, behavioral, and productive responses and thermal environment variables in laying hens reared in a semi‐arid region of Brazil. A total of 270 lightweight Hy‐Line White laying hens, 58 weeks old, with body weight of 1.60 ± 0.092 kg and egg production of 77.30% ± 3.62% were used. Simple Pearson correlation analysis showed fewer significant relationships than those identified by canonical correlation analysis. The results showed low to moderate canonical correlations (0.2576 ≤ rc ≤ 0.7449) between sets of indicators. Relationships between the thermal environment, thermoregulatory responses, and productive responses were significant (*p* ≤ 0.05), with the pair thermoregulatory responses × thermal environment presenting the highest correlation (rc = 0.7449; rc2 = 0.5548). Canonical correlation analysis is recommended to assess the behavior of complex relationships in laying hens. This multivariate approach provides a comprehensive understanding of linear and interactive relationships and captures interactions between climatic variables and thermoregulatory, behavioral, and productive responses.

## 1 Introduction

Recent IPCC assessments indicate that anthropogenic climate change has increased global temperatures by approximately 1.1°C relative to pre‐industrial levels, intensifying the frequency and severity of heat stress events. These changes pose a direct challenge to poultry production systems, particularly in tropical and semi‐arid regions, where elevated temperatures compromise bird welfare and productivity; for example, Silveira et al. (2026) reported increases of up to 40 beats per minute in respiratory rate of poultry under climate change scenarios projected for 2100, highlighting the physiological burden imposed by future thermal conditions.

The situation highlighted in the report creates a dilemma and underscores the need for studies aimed at understanding the influence of climate on the thermoregulatory physiology of farm animals, as well as the impact of heat stress on productive performance (de Castro Júnior et al. 2023; Silveira, Ferreira, et al. 2021; Silveira, de Vasconcelos, et al. 2025; Silveira, Ribeiro, et al. 2025). Considering that heat stress challenges global laying poultry production, where the ideal temperature ranges from 19°C to 22°C to ensure optimal performance (Pawar et al. 2016).

These birds are susceptible to heat stress due to increases in air temperature, which elevate metabolic demands, as birds are covered with feathers and lack sweat glands (Nawab et al. 2018). When laying hens are exposed to different levels of heat stress, they activate behavioral, physiological, and productive responses to dissipate sensible and latent heat in order to maintain thermoregulation and homeostasis (Kim et al. 2021). According to established animal welfare frameworks, such as those proposed by Dawkins (2017) and van der Staay et al. (2025), changes in physiological regulation, behavior, and productive performance are key welfare indicators, as they reflect the animal's ability to cope with environmental challenges and maintain biological functioning.

Most studies investigating the impact of the thermal environment on thermoregulatory responses use a univariate approach, particularly simple Pearson correlation analyses, to examine the relationship between physiological variables (Darji et al. 2024; Silveira, Silva, et al. 2021; Silveira et al. 2024). However, this study demonstrates that a comprehensive and multidimensional approach is essential to understand this relationship, given its multifactorial and intricate nature.

Multivariate analysis encompasses all statistical techniques that simultaneously examine multiple measurements in individuals or objects of study (Hair et al. 2014; Bodnara and Bodnar 2025). These methods constitute powerful analytical tools in several domains, enabling the simultaneous evaluation of multiple variables and their interactions (Silveira, Ribeiro, et al. 2025; Silveira, de Vasconcelos, et al. 2025). Therefore, they are advantageous when confronting data sets characterized by the involvement of numerous variables, each potentially exerting an influence on the others (Bodnara and Bodnar 2025).

Canonical correlation analysis (CCA), originally proposed by Hotelling (1936), is a multivariate technique designed to evaluate relationships between two sets of variables simultaneously. Rather than examining isolated associations, CCA allows the integration of environmental, physiological, behavioral, and productive indicators into a single analytical framework (Silveira, Ferreira, et al. 2021; Silveira, Ribeiro, et al. 2025). This approach is particularly relevant in animal physiology, where responses to stressors such as heat involve coordinated and interdependent mechanisms, making CCA a suitable tool for identifying integrated patterns of thermoregulatory and welfare‐related responses (Silveira, de Vasconcelos, et al. 2025).

The aim of this study was to compare the Pearson correlation method and CCA to understand and evaluate the relationship between thermoregulatory, behavioral, productive, and environmental thermal variables in laying hens reared in a semi‐arid region of Brazil. We hypothesized that CCA better represents the multidimensional relationships among thermal, behavioral, and productive variables in laying hens than traditional Pearson correlation.

## 2 Material and Methods

### 2.1 Experiment Location

The study was developed by combining two real databases obtained from the Laying Poultry Group of the *Universidade Estadual Vale do Acaraú* (UVA). The experimental period lasted three consecutive production cycles of 28 days each, occurring in the months of June, July, and August at the UVA Experimental Farm (FAEX), Sobral, Ceará, Brazil (3° 36″ S, 40° 18″ W and 56 m). The climate of the region is BSh according to the Köppen climate classification (Alvares et al. 2013). The regional annual mean temperature is 27°C, and the annual precipitation is 808 mm, concentrated in the first 5 months of the year (INMET, 2018).

Two hundred and seventy light‐weight laying hens of the Hy‐Line White lineage, 58 weeks old, body weight 1.60 ± 0.092 kg, and egg production of 77.30% ± 3.62%, were studied. All birds received the same management during the experimental period.

The birds were housed in galvanized wire cages of 90 × 45 × 45 cm dimensions, with three subdivisions of 30 cm and a population density of 450 cm^2^ per bird, containing a frontal feeder on a metal channel (Zatti, *Santa Catarina*, Brazil) and a drinking fountain (Nipple, Holandês, Brazil) in each cage in a masonry shed with East–West orientation, measuring 12 m long by 8 m wide and with a 2.60‐m ceiling height, wire mesh, concrete floor and ceramic tile cover. Drinking water was provided ad libitum. The lighting management of the facilities was 16 h/day, with 12 h of natural lighting and 4 h of artificial lighting at night. The experimental diets were formulated according to the recommendations of the Hy‐Line lineage manual (2016), and the chemical composition of the ingredients used in the formulation followed the recommendations of Rostagno et al. (2017). Diets were based on corn and soybean meal and met the nutritional requirements for laying hens, including metabolizable energy, crude protein, calcium, and available phosphorus, in accordance with Hy‐Line and NRC guidelines.

### 2.2 Data Collect

Although a total of 270 laying hens were used in the experiment (30 cages × 9 hens per cage), the unit of observation for the thermoregulatory and behavioral measurements was the individual hen selected per cage in each experimental cycle. In each cycle, one hen was randomly selected from the nine hens housed in each cage, resulting in 30 evaluated hens per cycle (one hen per cage). This procedure was repeated over three independent experimental cycles, totaling 90 individual observations (*n* = 90).

### 2.3 Thermal Environment

Dry‐bulb temperature (DBT,°C), wet‐bulb temperature (WBT,°C), and black globe temperature (BGT,°C) were recorded at 10‐min intervals from 06:00 a.m. to 5:00 p.m. using a weather station (TGD‐400, Instrutherm, São Paulo, Brazil) installed inside the experimental shed at a height of 80 cm above the floor. Sensors were calibrated according to the manufacturer's recommendations prior to data collection. Relative humidity (RH, %) was calculated from DBT and WBT data. For statistical analyses, measurements were averaged over the recording period for each day.

### 2.4 Thermoregulatory Responses

The thermoregulatory responses studied were: respiratory rate, cloacal temperature, thermal gradient, and surface temperature. The respiratory rate (RR, breaths per min) was observed for 15 s, and then the value was multiplied by four to obtain the movements per minute. Meanwhile, the cloacal temperature (TC,°C) was measured using a digital thermometer (accuracy ±0.1°C, G‐Tech, Rio de Janeiro, Brazil) inserted into the cloaca at a depth of 3 cm until temperature stabilized. The thermal gradient (temperature difference between the bird's body and the environment) was calculated by Equation (1), according to Castro et al. (2020).

(1) $$T_{G}=C_{T}-A_{T}$$

where TG is the thermal gradient (°C), CT is the cloaca temperature (°C), and AT is the air temperature (°C).

The surface temperature of different anatomical regions was measured using an infrared thermometer (accuracy ±0.1°C; STHT 77365, Stanley, USA) positioned 15 cm from the birds. Surface temperature of unfeathered body regions (TSSP,°C) was calculated as the mean temperature of the cloaca, crest, foot, and wattles, whereas surface temperature of feathered regions (TSCP,°C) was calculated from the mean temperatures of the wing, back, and head. This grouping was adopted because unfeathered regions play a major role in sensible heat dissipation, while feathered regions are more insulated and reflect core‐to‐surface heat transfer, as described in thermal mapping studies of poultry (Yahav and Plavnik 1999).

### 2.5 Behavioral Responses

The behavioral assessment was based on the ethogram of laying hen behavior described by Barbosa Filho et al. (2007), including sitting, eating, drinking, feather exploration, and pecking, and was visually assessed by five observers. Each observer received standardized technical training consisting of a 1‐h training session prior to data collection. Behavioral observations were recorded at consecutive 10‐min intervals during the morning and afternoon shifts, totaling 20 min of observation per hen per cycle. Intra‐observer reliability was evaluated prior to the experimental period using Cohen's kappa coefficient, yielding values ≥ 0.80, which is considered acceptable agreement. Table 1 presents the behavioral ethogram used in this study.

**TABLE 1:** Behavioral ethogram of commercial laying hens reared in a conventional cage system.

Behaviors	Description	Events
Sitting	When the lower dorsal part of the bird is in contact with the cage	Frequency
Eating	Act in which the bird is eating food from the feeder	Frequency
Exploring Feathers	The act of exploring warping as a form of investigation whether it is theirs or not	Frequency
Pecking	Act in which the bird pecks in the cage, on the sides, on the floor, feeder or drinker, aggressively or not, as a form of exploration.	Frequency

### 2.6 Productive Responses

The productive responses studied were: feed intake (g/bird/day), egg production (%) and egg mass conversion (kg/kg), during the production cycle (28 days). Feed intake (g/bird/day). Performance was measured daily throughout the experimental period.

### 2.7 Databases

The database used in this study was processed in two sequential stages. The original dataset comprised 18 variables (Database 1; *n* = 90), grouped into thermal environment (air temperature and relative humidity), thermoregulatory responses (thermal gradient, surface temperature with feathers, surface temperature without feathers, cloacal temperature, and respiratory rate), behavioral responses (sitting, eating, drinking, pecking, and feather exploration), and productive responses (feed intake, egg production, feed conversion per egg mass, and feed conversion per dozen eggs).

In the first stage, variable selection was performed to reduce multicollinearity and remove redundant or weakly informative predictors prior to multivariate modeling. Pairwise Pearson correlation coefficients were calculated using SPSS (version 20), and variables showing strong collinearity (|r| ≥ 0.70) were screened. When two or more variables were highly correlated, the variable with lower biological interpretability or weaker association with the response variables was excluded, based on exploratory correlation matrices and prior biological relevance. This procedure resulted in a reduced set of 13 variables (Database 2; *n* = 90).

In the second stage, exploratory data analysis was conducted in R, and potential outliers were identified using boxplots and standardized scores. Observations with standardized values exceeding ±3 standard deviations from the mean were considered extreme and removed, following common practice in physiological and behavioral datasets to minimize undue leverage of extreme values. This procedure resulted in the final dataset (Database 3; *n* = 88).

## 3 Statistical Methods

In describing the methodology, it was necessary to define some statistical properties. The explanation provided will follow the framework outlined by Johnson and Wichern (1986).

This technique is based on the correlation between a linear combination of a set of dependent variables ($X_{p}$) and a linear combination of another set of independent variables $\left(W_{q}\right)$. Linear combinations of variables are very useful for comparison and prediction (Johnson and Wichern 2007). Thus, linear combinations of sets of variables can be defined as follows:

(2) $$U_{i}=a_{i1}X_{1}+a_{i2}X_{2}+\ldots a_{\mathrm{ip}}X_{p}$$

(3) $$V_{i}=b_{i1}W_{1}+b_{i2}W_{2}+\ldots b_{\mathrm{iq}}W_{q}$$

where $a_{\mathrm{ip}}$ and $b_{\mathrm{iq}}$ are canonical coefficients.

$U_{i}$ and $V_{i}$ are the *i*th pair of canonical variables.

$U_{1}$ and $V_{1}$ form the first pair of canonical variates which is associated to the first canonical correlation, as follows:

(4) $$r_{1}=\frac{C\hat{o}v\;\left(U_{1},V_{1}\right)}{\sqrt{\;Vân\;\left(U_{1}\right)\;Vân\;\left(V_{1}\right)}}$$

The percentage of variance explained by the first canonical variates is $U_{\mathrm{Xi}}^{2}$ e $V_{\mathrm{Wi}}^{2}$ is

(5) $$U_{\mathrm{Xi}}^{2}=\frac{\sum_{j=1}^{p}a_{\mathrm{ij}}^{2}}{p}\;\text{and}\;V_{\mathrm{Wi}}^{2}=\frac{\sum_{j=1}^{q}b_{\mathrm{ij}}^{2}}{q}$$

where

*p* is number of variables of *X*.

*q* is number of variables of *W*.

The total number of pairs of canonical variates is defined by the minimum value between *p* and *q*.

Finally, the CCA produced a set of new variables called the canonical functions (*C*_*F*_), which are linear combinations of the original variables, as reported in the following equation:

(6) $$C_{F}=d_{1}X_{1}+d_{2}X_{2}++d_{3}X_{3}+\ldots d_{n}X_{n}$$

where $d_{n}$ are the canonical coefficients which indicate the contribution of each variable in the composition of *C*_*F*_.

X are the scores of the *n* original variables.

As the data in question were on different scales, *z*‐score standardization was applied, which consists of standardizing the variables to a mean of 0 and a standard deviation of 1. In order to achieve this result, the *scale function* was applied through the R Studio software, with the aim of transforming the data so that all variables are comparable with each other, regardless of their units of measurement or amplitude.

Table 3 shows the identification of the dependent and independent variables for the six canonical correlations calculated. It is important to highlight that the first canonical function was considered for all canonical correlations under study, as this captures the maximum possible correlation between two sets of variables.

Furthermore, synthetic variables were defined as those that are not directly observed, but rather artificially created from other variables. In the context of CCA, canonical functions are synthetic variables that represent linear combinations of the original variables from two different data sets, maximizing the correlation between these sets.

The canonical correlation coefficient ($r_{c}$) represents the Pearson correlation between two synthetic variables within a given canonical function. It quantifies the linear relationship between pairs of canonical variables derived from two sets of variables. The magnitude of the correlation coefficient reflects the strength of the linear association between the canonical variables. A coefficient close to +1 or −1 indicates a strong positive or negative linear relationship, respectively, while values close to 0 suggest a weak linear relationship. The sign of the correlation coefficient denotes the direction of the linear relationship between the canonical variables: A positive coefficient implies that high values of one variable correspond to high values of the other variable, while a negative coefficient suggests an inverse relationship. Conversely, a low correlation or a correlation close to zero may imply a limited association between the two sets of variables.

The squared canonical correlation coefficient ($r_{c}^{2}$) quantifies the extent of shared variance between pairs of canonical variates derived from two sets of variables. It assesses the extent to which the variability in one set of canonical variates can be explained by the variability in the other set. A higher squared canonical correlation indicates that a greater proportion of the variability in one set of canonical variates is accounted for by the other set. Analogous to Pearson correlation coefficients, the magnitude of the squared canonical correlation means the intensity of the relationship between pairs of canonical variates. A squared canonical correlation close to 1 denotes a robust linear relationship between the canonical variates, while a value close to 0 means a weak relationship.

In summary, while the canonical correlation ($r_{c}$) measures the strength and direction of the linear association between sets of canonical variables, the canonical coefficient of determination ($R_{c}^{2}$) provides a more descriptive measure of the proportion of variability explained between sets of canonical variables.

In addition to interpreting the magnitude and direction of correlation coefficients and squared canonical correlations, it is essential to assess their statistical significance. A commonly used method to assess significance in CCA is through Wilks' lambda, which is a multivariate statistic test used to assess the overall significance of canonical correlations in CCA. It tests the null hypothesis that there is no relationship between the sets of variables represented by the canonical variables. A significant result indicates that at least one canonical correlation is nonzero, implying a significant relationship between the sets of variables.

Hypothesis testing with Wilks' lambda determines whether the observed squared canonical correlations reach statistical significance at a specified level. In this study, the significance level adopted was 0.05. A significant *p* value rejects the null hypothesis, supporting the presence of a significant relationship between the sets of variables, which ensures that the observed relationships are not merely coincidental and increases confidence in the interpretation of the results. All analyses were conducted in RStudio 2023.09.1 using the “CCA” package.

## 4 Results and Discussion

**FIGURE 1:**
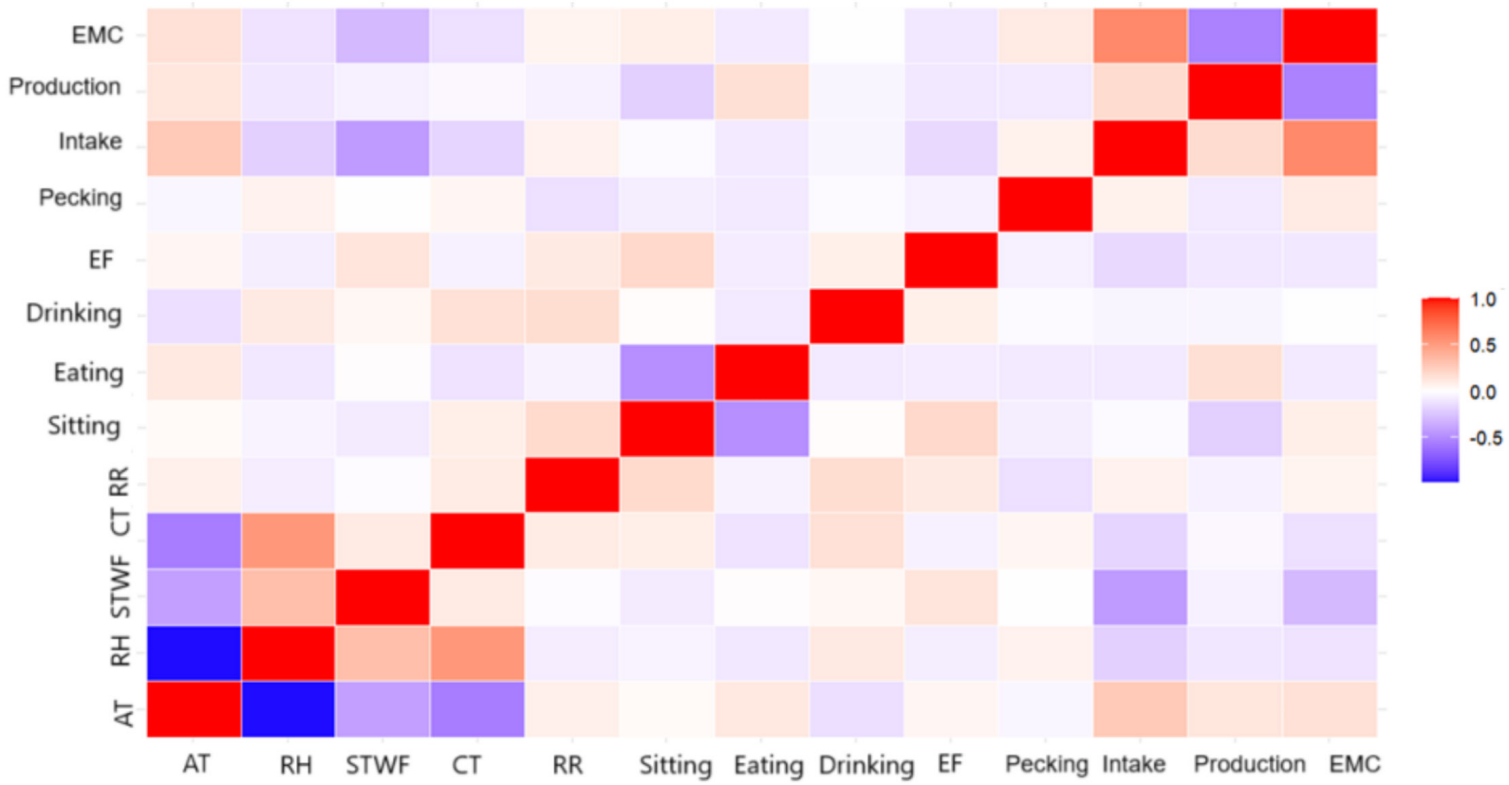
Heat map with Pearson's correlation matrix for the variables that make up the thermal environment, thermoregulatory responses, behavioral responses, and productive responses for laying hens. AT, air temperature (°C); CT: cloacal temperature (°C); EF, exploiting feather; EMC, egg mass conversion (kg/kg); PRO., production (%); RH, relative humidity (%); RR, respiratory rate (breaths/min), intake (g/birds/day); STWF, surface temperature with feathered (°C).

Initially, for all variables selected in Database 3, calculations were performed to determine the minimum and maximum values, first and third quartiles, arithmetic mean, median, and standard deviation (Table 2) and to obtain the Pearson correlation matrix through the heat map (Figure 1).

**TABLE 2:** Descriptive statistics for thermal environment, thermoregulatory responses, behavioral responses, and productive responses for laying hens.

Indicators	Variables	Min.	1st quarter	Median	Average	±SD	3rd quarter.	Max.
Thermal environment	Air temperature (°C)	30.55	30.55	31.67	31.42	0.64	32.05	32.05
	Relative humidity (%)	46.83	46.83	47.30	49.28	3.15	53.70	53.70
Thermoregulatory responses	Feathered surface temperature (°C)	29.95	32.11	32.72	32.64	0.91	33.18	35.02
	Cloacal temperature (°C)	40.55	41.01	41.20	41.19	0.25	41.39	41.85
	Respiratory rate (breaths/min)	24.00	60.00	72.00	70.89	20.62	82.00	120.00
Behavioral responses (frequency of behavior/min)	Sitting	2.00	6.25	9.00	9.80	4.67	13.00	21.00
	Eating	16.00	23.00	27.50	27.86	6.30	33.00	46.00
	Drinking	1.00	3.25	4.00	4.73	1.91	6.00	10.00
	Feather exploration	0.00	1.00	2.00	2.60	1.85	4.00	8.00
	Pecking	0.00	1.00	2.00	2.00	1.76	3.00	9.00
Productive responses	Intake (g/bird/day)	83.40	93.38	95.72	95.51	1.60	97.83	110.67
	Production (%)	66.27	75.00	76.39	76.49	3.92	78.97	86.51
	Conversion by egg mass (kg/kg)	1.70	1.91	1.99	1.99	0.11	2.05	2.28

Abbreviations: 1st Qu, first quartile; 3rd Qu, third quartile; min, minimum; max, maximum; SD: standard deviation.

When assessing the thermal environment of the laying hens, the average air temperature (31.42°C) combined with a relatively low relative humidity (49.28%) was clearly outside the recommended comfort ranges for laying hens (19°C–22°C and 50%–60%), characterizing a hot and dry environment (Romijin and Lokhorst 1966; Pawar et al. 2016; Kim et al. 2022). Under these conditions, rapid evaporative heat loss predominates, which may lead to dehydration and skin irritation, thereby compromising thermoregulatory efficiency and inducing physiological adjustments such as increased respiratory rate and changes in surface and cloacal temperatures (Kim et al. 2022). From an animal‐welfare perspective, this thermal challenge is expressed through stress‐related behavioral responses, including increased panting, reduced feed intake, and alterations in normal activity patterns, with subsequent negative impacts on productive performance, such as reduced egg size, body weight gain, eggshell quality, and increased water consumption (Kursun 2019; Kuchmistov 2022).

It is possible to observe that in Figure 1, the thermal environment has a strong relationship between the variables. Variables related to thermoregulatory responses showed low linear correlations (|*r*| < 0.5) among themselves, whereas within the behavioral responses, the highest correlation was observed between *sitting* and *eating*. In the productive responses' matrix, *egg mass conversion* showed moderate linear correlations with *intake* (*r* = 0.60) and *production* (*r* = −0.54). Correlation strength was interpreted based on predefined thresholds, with |*r*| ≥ 0.5 considered moderate to strong associations.

After obtaining descriptive statistics, CCA was applied. This involved estimating the canonical correlation coefficient to determine whether the relationships between the selected variables were significant. Attention is focused on the measures of association between two groups of variables, delineated as a linear combination of one set of dependent variables ($X_{p}$) and a linear combination of another set of independent variables ($Y_{q}$).

Table 3 shows that the canonical correlation coefficient is higher between sets of variables compared with the Pearson correlation coefficient between two variables (Figure 1). This result shows that linear combinations are more strongly associated than any pair of individual variables, and it also highlights the importance of considering the multidimensional structure of the data to understand the complexity of the associations between them (Thompson 1991).

**TABLE 3:** Canonical correlations between the thermal environment, thermoregulatory, behavioral and productive responses of laying hens, as well as the *p* value of the Wilks' lambda test for these indicators.

Dependent variables	Independent variables	Canonical *R* $\left(r_{c}\right)$	$r^{2}$ canonical $\left(R_{c}^{2}\right)$	*p*
Thermoregulatory responses	Thermal environment	0.7449	0.5548	0.0000
Behavioral responses	Thermal environment	0.2576	0.0663	0.5288
Productive responses	Thermal environment	0.5048	0.2548	0.0003
Behavioral responses	Thermoregulatory responses	0.3050	0.0930	0.5269
Productive responses	Thermoregulatory responses	0.4615	0.2130	0.0133
Behavioral responses	Productive responses	0.3017	0.0910	0.6240

Several studies have investigated adaptive responses using simple Pearson correlation analyses (Čukić et al. 2023; Silveira, Silva, et al. 2021; Silveira et al. 2024). However, because Pearson's correlation assumes linear relationships and does not account for interactions among multiple variables, it is limited in capturing the multifactorial nature of animal adaptation (Yu and Jia 2024). In contrast, CCA allows the simultaneous evaluation of interconnected physiological, behavioral, and environmental variables, providing a more integrative view of adaptive responses. This multivariate approach enables the detection of coordinated stress–response patterns that are not evident in univariate models, thereby improving the assessment of animal welfare under challenging thermal conditions.

Table 3 shows low to moderate canonical correlations (0.2576 ≤ $r_{c}$ ≤ 0.7449) and squared canonical correlations (0.0910 ≤ $r_{c}^{2}$ ≤ 0.5548) among the indicator sets. Although statistically significant according to Wilks' lambda (*p* ≤ 0.05), these correlations also have clear biological meaning. In particular, the strong association between thermoregulatory responses and the thermal environment ($r_{c}$ = 0.7449; $r_{c}^{2}$ = 0.5548) indicates that more than half of the shared variance between these sets is explained by environmental conditions, reflecting the central role of ambient temperature and humidity in shaping physiological adjustments. This result highlights that changes in the thermal environment are closely linked to coordinated thermoregulatory responses, with direct implications for productive performance and animal welfare under heat stress.

**FIGURE 2:**
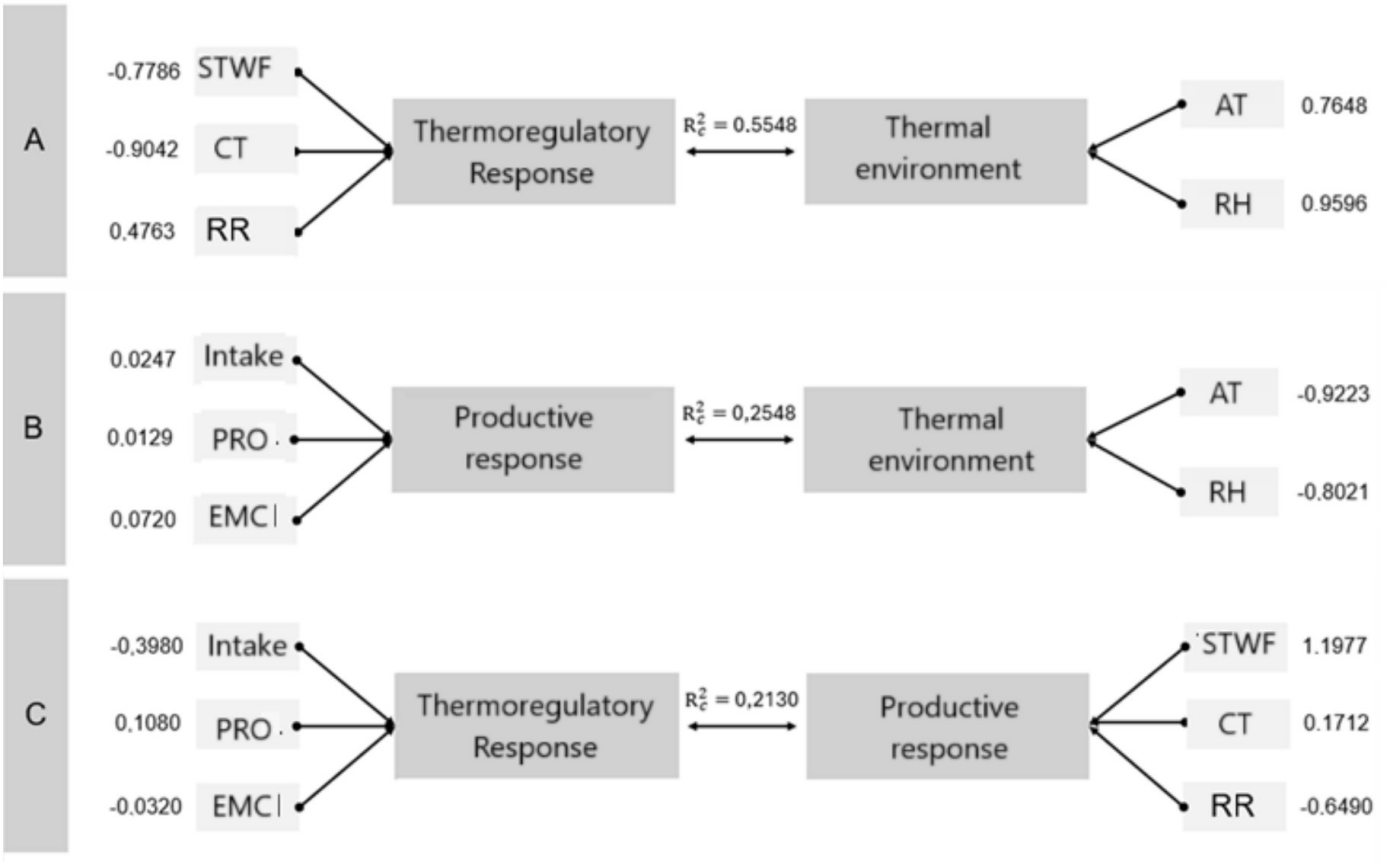
Graphical representation of the first canonical function for each pair of canonical variables for the relationship between the indicators under study (*p* value ≤ 0.05). AT, air temperature (°C); CT, cloacal temperature (°C); EMC, egg mass conversion (kg/kg); PRO., production (%); RH, relative humidity (%); RR, respiratory rate (breaths/min), intake (g/birds/day); STWF, surface temperature with feathered (°C).

For better visualization of the significant canonical correlations, a graphical representation of the standardized canonical loads was made for each pair of canonical variables studied (Figure 2). The canonical correlation between the thermal environment and the thermoregulatory responses was significant (*p* = 0.0000) and presented $r_{c}$ = 0.7449 and $r_{c}^{2}$ = 0.5548. The thermal environment variables presented high canonical loads, influencing the indicators of the thermoregulatory responses; therefore, the cloacal temperature and surface temperature with feathers were the most impacted, respectively. On the other hand, the respiratory rate (RR) presented a low canonical load. This result allows us to infer that air temperature and relative humidity have a greater effect on the cloacal temperature of the laying hens. This indicator reflects how the bird is managing its heat internally, as well as on the surface temperature with feathers (mean temperature of the wing, back, and head). These responses illustrate physiological prioritization under heat load (Nawab et al. 2018; Kim et al. 2021).

The canonical correlation between the thermal environment and the productive responses was significant (*p* = 0.0003) and presented $r_{c}$ = 0.5048 and $r_{c}^{2}$ = 0.2548. The productive responses presented low canonical loads, which shows that they were influenced slightly by the thermal environment variables.

The canonical correlation between the thermoregulatory responses and the productive responses was significant (*p* = 0.0133) and presented $r_{c}$ = 0.4615 and $r_{c}^{2}$ = 0.2130. This indicates that approximately 21% of the shared variance between these response sets is explained by their association. The relatively low canonical loadings of most thermoregulatory variables suggest a limited overall influence on productive traits; however, surface temperature of feathered regions showed a stronger contribution, indicating its relevance as a thermal indicator linked to production. These findings are consistent with previous reports under chronic heat exposure, in which productive performance is only moderately affected by thermoregulatory adjustments (Kim et al. 2021).

CCA provides a powerful multivariate framework for examining interactions among thermal, behavioral, and productive indicators in laying hens. This approach captures the complexity of welfare‐related responses under heat stress and can support precision management strategies in poultry production.

## Author Contributions

Carolina de Oliveira Marques de Souza: writing – original draft and statistical analysis. Silvana Cavalcante Bastos Leite: supervision, project administration, methodology, and funding acquisition. Maria Rogervânia Silva de Farias: data collection, and final review. Angela Maria de Vasconcelos: supervision, project administration, and methodology. Luiz Paulo Fávero: statistical analysis, final review, and validation.

Aérica Cirqueira Nazareno: writing – original draft and final review. Laura Bertolaso de Vecchi: conceptualization, final review, and validation. Robson Mateus Freitas Silveira: conceptualization, writing – original draft, validation, statistical analysis, and supervision.

## Funding

The authors received no specific funding for this work.

## Ethics Statement

All procedures performed were approved by the Animal Use Ethics Committee (CEUA) of the Vale do Acaraú State University (UVA), Ceará, Brazil (Protocol N^o^. 002.02.018. UVA.504.03).

## Conflicts of Interest

The authors declare no conflicts of interest.

## Artificial Intelligence Statement

The authors declare that ChatGPT was used only to assist with language editing and to improve clarity and readability during the preparation of this manuscript. All scientific content, analyses, interpretations, and conclusions were developed and verified by the authors, who take full responsibility for the final version of the manuscript.

## Data Availability

The datasets used and/or analyzed during the current study are available from the corresponding author on reasonable request.
